# Functional Reintegration of Sensory Neurons and Transitional Dendritic Reduction of Mitral/Tufted Cells during Injury-Induced Recovery of the Larval *Xenopus* Olfactory Circuit

**DOI:** 10.3389/fncel.2017.00380

**Published:** 2017-11-28

**Authors:** Sara J. Hawkins, Lukas Weiss, Thomas Offner, Katarina Dittrich, Thomas Hassenklöver, Ivan Manzini

**Affiliations:** ^1^Institute of Neurophysiology and Cellular Biophysics, University of Göttingen, Göttingen, Germany; ^2^Institute of Animal Physiology, Department of Animal Physiology and Molecular Biomedicine, Justus Liebig University Giessen, Giessen, Germany; ^3^Center for Nanoscale Microscopy and Molecular Physiology of the Brain (CNMPB), Göttingen, Germany

**Keywords:** *Xenopus laevis*, olfactory receptor neurons, neuronal stem cells, degeneration, regeneration, glomerulus, network reconstruction

## Abstract

Understanding the mechanisms involved in maintaining lifelong neurogenesis has a clear biological and clinical interest. In the present study, we performed olfactory nerve transection on larval *Xenopus* to induce severe damage to the olfactory circuitry. We surveyed the timing of the degeneration, subsequent rewiring and functional regeneration of the olfactory system following injury. A range of structural labeling techniques and functional calcium imaging were performed on both tissue slices and whole brain preparations. Cell death of olfactory receptor neurons and proliferation of stem cells in the olfactory epithelium were immediately increased following lesion. New olfactory receptor neurons repopulated the olfactory epithelium and once again showed functional responses to natural odorants within 1 week after transection. Reinnervation of the olfactory bulb (OB) by newly formed olfactory receptor neuron axons also began at this time. Additionally, we observed a temporary increase in cell death in the OB and a subsequent loss in OB volume. Mitral/tufted cells, the second order neurons of the olfactory system, largely survived, but transiently lost dendritic tuft complexity. The first odorant-induced responses in the OB were observed 3 weeks after nerve transection and the olfactory network showed signs of major recovery, both structurally and functionally, after 7 weeks.

## Introduction

While many mammalian species appear to have lost regenerative capacity of neuronal tissue during evolution, early diverging vertebrates exhibit elevated neuroregenerative potential and have been shown to be capable of restoring entire brain regions after lesion (for review Ferretti, 2011). This ability is not only variable among species but also has a strong developmental component. After development is complete, most stem cells of the central nervous system undergo terminal differentiation and lose their ability to divide (Kauffman, 1968; Caviness et al., 1995). Even in cases where neurogenesis is still possible, the system often no longer possesses the mechanisms that once allowed newly formed neurons to successfully integrate into a neural circuit (Christie and Turnley, 2013).

The vertebrate olfactory system is an exceptional case and has become increasingly more relevant as a model to study neuroregenerative processes, as it is known for its lifelong capacity to replenish cells lost during natural turnover (Graziadei and Metcalf, 1971; Graziadei, 1973), as well as to regenerate after severe lesion (Schwob, 2002). Olfactory receptor neurons (ORNs) of the olfactory epithelium reside in an exposed location, prone to external stress factors, and thus have a limited lifespan. Eventually they undergo caspase-mediated programmed cell death and multipotent stem cells of the basal layers of the olfactory epithelium compensate for this loss in order to sustain the sense of smell (Cowan and Roskams, 2002; Leung et al., 2007). Newly generated sensory neurons extend their axons toward the olfactory bulb (OB) in search of synaptic targets, eventually forming functional connections with second order neurons, mitral and tufted cells, in functional structures called glomeruli (Nezlin et al., 2003; Manzini, 2015; Kosaka and Kosaka, 2016). A correct integration is essential to allow the successful propagation of olfactory information from second order neurons to higher brain centers. Not only input neurons of the olfactory network, but also neurons of the odor processing brain circuitry, are constantly replaced (Kaplan and Hinds, 1977). The stem cell population located in the subventricular zone is mainly responsible for supplying the OB with new interneurons (Lim and Alvarez-Buylla, 2016), which has been shown to be essential in adjusting olfactory performance (Mouret et al., 2009).

The turnover of neurons that occurs both in the olfactory epithelium and OB is not restricted to developmental stages, but continues throughout life, allowing network adaptations to sensory experience, learning, and even recovery after extensive injury (Schwob, 2002; Brann and Firestein, 2014; Cummings et al., 2014). Although the regenerative capacity of the olfactory system has been illustrated numerous times in various organisms, the way in which this system is capable of withstanding massive lesion and recovering functionally is still not fully understood (Yu and Wu, 2017). It is crucial to understand the processes that hinder an efficient recovery, and to find perspectives to facilitate correct network reconstitution. Olfactory dysfunction following head trauma is common, and is frequently due to shearing injuries at the cribriform plate that lacerate the olfactory nerves (ONs; Coelho and Costanzo, 2016). Recovery is variable and highly dependent on the severity and location of the injury (Doty et al., 1997; Gudziol et al., 2014; Coelho and Costanzo, 2016). Increased inflammation and glial scar formation that occur in the mammalian system pose a major challenge for ON recovery and successful reinnervation of the bulbar network by axons of newly generated ORNs (Kobayashi and Costanzo, 2009). After loss of olfactory sensory input in mammals, it has been shown that mitral cell dendritic tufts persistently degrade (Murai et al., 2016). In humans, only approximately 1/3 of patients show at least a partial recovery of their sense of smell (Doty et al., 1997; Gudziol et al., 2014) and no effective treatment is available yet to treat post-traumatic neuronal damage or to assist in the recovery of the olfactory system (Coelho and Costanzo, 2016).

**Figure 1:**
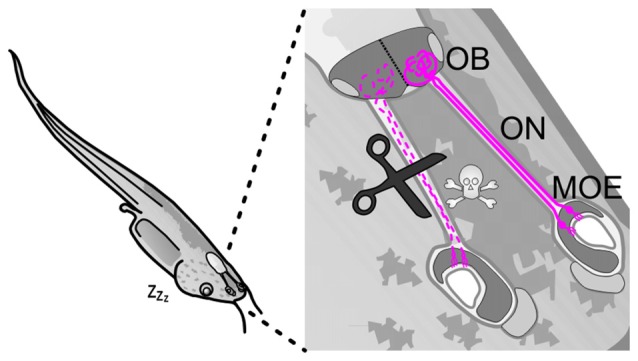
Olfactory nerve transection as a model injury to induce neuronal damage in the olfactory system of larval *Xenopus laevis*. Schematic depiction of a tadpole with a close up of its olfactory system. Bipolar olfactory receptor neurons (magenta) of the MOE extend their axons via the ON into the OB. Fine scissors can be used to transect the ON, leading to axon degeneration and olfactory receptor neuron cell death. MOE, main olfactory epithelium; OB, olfactory bulb; ON, olfactory nerve.

We performed ON transection on larval *Xenopus laevis* in order to disrupt the neuronal network of a highly regenerative vertebrate olfactory system (Figure 1). The aim was to further understand aspects of degeneration and recovery of neural circuits after injury, and to investigate how neural disruption and the potential for circuit restoration in this system differs from that found in the mammalian system. We show that ON transection targets ORNs for cell death, leaving other components of this system involved in the process of regeneration largely unperturbed. We have established a timeline of post-transection events, up until the point of recovery of the olfactory system, revealing a transient decline of dendritic arborizations of postsynaptic mitral/tufted cells (MTCs) during the period of denervation. Our results are a clear illustration of how the maintenance of a permissive environment in a highly regenerative system can allow neuronal regeneration and subsequent formation of correct axonal and dendritic connections, creating a reliable foundation for future research on the topic of neuroregeneration.

## Materials and Methods

### Animal Care, Olfactory Nerve Transection and Sensory Neuron Labeling

All *Xenopus laevis* larvae used in this study were raised in our breeding colony at the University of Göttingen. They were kept in water tanks (50 l) at a water temperature of 19–22°C and fed with algae (Dose Aquaristik). All procedures for animal handling were approved by the governmental animal care and use office (Niedersächsisches Landesamt für Verbraucherschutz und Lebensmittelsicherheit, Oldenburg, Germany, Az.16/2136) and were in accordance with the German Animal Welfare Act as well as with the guidelines of the Göttingen University Committee for Ethics in Animal Experimentation. As an injury model for substantial damage in the olfactory system, we transected the ONs of *Xenopus laevis* tadpoles to disrupt the neuronal population in the olfactory organ. For ON transection, we used freely swimming, premetamorphic larvae with an already well developed olfactory system, ranging from developmental stage 48 (ca. 7 days after fertilization at 22–24°C) to stage 51 (ca. 17 days after fertilization at 22–24°C; Nieuwkoop and Faber, 1994). We surveyed the extent of recovery only in animals that did not exceed developmental stage 56, when major metamorphic remodeling of the olfactory system started to occur. Pigmented and albino *Xenopus laevis* larvae were anesthetized in 0.02% MS-222 (ethyl 3-aminobenzoate methanesulfonate; Sigma-Aldrich), and their ONs were transected with very fine scissors without damaging surrounding tissue (Figure 1). To label sensory neurons, Biocytin (ε-biotinoyl-L-lysine, Molecular Probes, ThermoFisher Scientific) or microRuby crystals (tetramethylrhodamine/biotin linked dextran, 3 mM; Molecular Probes, Thermo Fisher Scientific) were placed into the lesioned nerve in immunohistochemistry experiments and in experiments to visualize axonal degradation in the OB (see below), respectively. The wound was closed with tissue adhesive (Histoacryl L; Braun). After transection, animals were transferred to a beaker filled with fresh tap water for recovery. In a subset of experiments, this transection procedure was repeated every week to survey the volumetric changes in the OB (see below). At different time intervals after injury, animals were chilled in ice water until paralyzed and killed by severing the brain and spinal cord with a scalpel. Subsequent experiments were performed on an excised block of tissue containing the olfactory epithelia, ONs and the OB.

### Immunohistochemistry

To visualize changes in the olfactory epithelium and OB after bilateral ON transection we performed immunolabeling on slices of the olfactory system. For 5-bromo-2′-deoxyuridine (BrdU, Sigma-Aldrich) labeling experiments, animals were kept in normal tap water with 100 μM BrdU for 24 h before dissection. BrdU exposure with this concentration was shown to not induce negative side effects, such as increased apoptosis, alterations of cell marker expression patterns or foraging behavior (Raices et al., 2015). Animals were killed (as described above) 1,2,3,7 and 21 days after ON transection (as described above). Seven and 21 days post-transection, newly generated ORNs were labeled via an additional ON transection 1 h before killing the animals. Excised tissue blocks were fixed in 4% formaldehyde, washed in PBS, embedded in 5% low-melting-point agarose (Sigma-Aldrich), glued onto the stage of a vibratome (VT 1200S, Leica) and cut horizontally into slices. Tissue blocks used to label the olfactory epithelium were sliced at 75 μm thickness, whereas tissue used to label the OB was cut at 95 μm thickness. Nonspecific binding was blocked with 2% normal goat serum (NGS; MP Biomedicals) in PBS containing 0.2% Triton X-100 (PBST, Carl Roth) for 1 h. Slices of BrdU treated animals were incubated in 1 N HCl at 37°C for 45 min to denature DNA and subjected to multiple changes of PBS. Slices were incubated overnight at 4°C with one of the following primary antibodies—anti-*Xenopus laevis* cytokeratin II (1h5, monoclonal, derived from mouse, obtained from the Developmental Studies Hybridoma Bank developed under the auspices of the National Institute of Child Health and Human Development and maintained by the University of Iowa, Department of Biological Sciences (Iowa City, IA)); anti-active caspase-3 (polyclonal anti-active caspase-3, ab13847, derived from rabbit using a synthetic peptide corresponding to human active + procaspase 3 aa 150–250 conjugated to keyhole limpet hemocyanin (KLH), RRID:AB_443014; Abcam; characterized by Thompson and Brenowitz (2010) and previously used in *Xenopus laevis* tissue by Dittrich et al. (2016)); or anti-BrdU (B2531, monoclonal, derived from mouse, Sigma-Aldrich). Primary antibodies were diluted in 2% NGS/PBST (1:1000, 1:600, 1:100 respectively) and washed off with PBS after the incubation period. Alexa 488-conjugated goat anti-mouse or anti-rabbit secondary antibody (Invitrogen, Thermo Fisher Scientific) was applied at a dilution of 1:250 in 1% NGS/PBS for 1 h. The secondary antibodies were washed off in several changes of PBS. To visualize biocytin-backfilled ORNs, slices were incubated in Alexa 568- or 647-conjugated streptavidin (Molecular Probes, Thermo Fisher Scientific) at a final concentration of 5 μg/ml in PBST for 1 h and repeatedly rinsed in PBS. In active caspase-3 immunostaining experiments, cell nuclei were labeled with 10 μg/ml propidium iodide (Molecular Probes, Thermo Fischer Scientific) for 15 min. After repeated washing steps with PBS, slices were mounted on glass slides using mounting medium (Dako). Images stacks were acquired using upright confocal laser-scanning microscope (LSM 780/Axio Examiner, Zeiss) with an axial resolution of 1 μm.

### Whole Mount Preparation of the Olfactory Bulb

After excising a tissue block containing the olfactory system as described above, the ventral palatial tissue was removed to expose the ventral side of the OB and the caudal portion of the ONs. This whole mount preparation was transferred into an imaging chamber containing standard bath solution (see “Solutions” section) and stabilized by a small platinum grid with nylon threads.

### Visualization of Axonal Debris in the Olfactory Bulb

Animals were killed and whole mount preparations of the OB were made from excised tissue blocks 1, 2, 3 and 7 days after unilateral ON transection (as described above). Microruby labeled, degenerating ORN axons were visualized from the ventral side of the OB using an upright multi-photon microscope (A1R-MP, Nikon).

### Olfactory Receptor Neuron Labeling Via Electroporation

To visualize axonal reinnervation of the OB, fluorophore-coupled dextran (Alexa 594, Alexa 488, Cascade Blue, 10,000 MW, Molecular Probes, Thermo Fisher Scientific) was introduced into sensory neurons via electroporation (for details, see Haas et al., 2002; Hassenklöver and Manzini, 2014). Albino *Xenopus* larvae were anesthetized, dye crystals were introduced into both nasal cavities and dissolved in the residual moisture. Two thin, platinum electrodes were carefully placed in the nasal cavities. The electrodes were connected to a voltage pulse generator (ELP-01D; npi Electronics), and 12 pulses (20–25 V, 25 ms duration at 2 Hz) with alternating polarity were applied. After electroporation, animals were transferred into a beaker filled with fresh tap water for recovery and killed 1 or 2 days later. Whole mount preparations of the OB were made from excised tissue blocks 1, 2, 3 and 7 weeks after unilateral ON transection (as described above). Image stacks of the whole intact OB were acquired from the ventral side (for details see Hassenklöver and Manzini, 2014) using an upright multi-photon microscope (A1R-MP, Nikon).

### Labeling of the Olfactory Bulb for Volume Quantification

Animals were killed (see above) 1, 2, 3 and 7 days after unilateral nerve transection and a whole mount preparation of the OB was prepared from excised tissue blocks (as described above). The preparation was incubated in bath solution (see “Solutions” section) containing 100 μM Calcein green/AM (Molecular Probes, Thermo Fisher Scientific), 30 μM Alexa 594 dextran (10,000 MW, Molecular Probes, Thermo Fisher Scientific), and 50 μM MK571 (Sigma-Aldrich), an inhibitor of multidrug resistance transporters. Calcein green/AM was initially dissolved in DMSO (Sigma-Aldrich) and Pluronic F-127 (Molecular Probes, Thermo Fisher Scientific). After an incubation time of 2 h, a series of image stacks of the whole ventral OB was obtained using multiphoton microscopy. Relative changes of the OB volume of the transected side to the non-transected side were compared.

### Sparse Cell Electroporation to Label Individual Mitral/Tufted Cells

Individual MTCs were investigated for morphological changes after the loss of ORN axon innervation. Animals were killed 1, 3 and 7 weeks after unilateral nerve transection and whole mount preparations were produced from tissue blocks containing the olfactory system (as described above). MTCs were labeled via sparse cell electroporation in the ventrolateral OB using a stereomicroscope with epifluorescence illumination. Electroporation micropipettes (Warner instruments, resistance 10–15 MΩ) were filled with Alexa 488 or Alexa 594 dextran solution (1–5 μl, 3 mM dissolved in bath solution) and mounted on a pipette holder containing a silver wire electrode covered with silver chloride. A train of square voltage pulses (50 V, 300 μs, 500 ms at 200–300 Hz) was triggered by an Axoporator 800A (Molecular devices) single cell electroporator to transfer dye into neurons (for more details, see Hassenklöver and Manzini, 2014). Image stacks of MTCs were acquired with a z-resolution of 1–2 μm using multiphoton microscopy from the ventral side of the OB. MTCs labeled in the non-transected side of the OB were used as controls.

### Calcium Imaging Experiments

To analyze functional changes in the various cell populations of the main olfactory epithelium (MOE) after ON transection, functional calcium imaging experiments were performed on acute slices of the olfactory organ. Tissue blocks containing the olfactory system were glued to the stage of a vibratome. Two horizontal cuts were made to produce a slice with cells of the MOE exposed on both the dorsal and ventral side. Depending on angular position of the tissue block and size of the olfactory organ, the final slice thickness was between 120 μm and 140 μm. Calcium indicator mixture consisted of 50 μM Fluo-4/AM (Molecular Probes, Thermo Fisher Scientific), 100 μM MK571 (Sigma-Aldrich) in bath solution. Fluo-4/AM was initially dissolved in DMSO and Pluronic F-127 with final concentrations not exceeding 0.5% and 0.1%, respectively. Slices were incubated for 35 min with calcium indicator mixture on a shaker. Changes of intracellular calcium concentration of individual cells of the MOE were monitored using a laser-scanning confocal microscope (LSM 510/Axiovert 100 M, Zeiss, Jena, Germany). A time series of one focal plane was acquired. Virtual slice thickness excluded fluorescence from more than one cell layer and the field of view covered a square with an edge length of 360 μm.

We performed calcium imaging in whole mount olfactory system explants to investigate the extent of functional recovery of the olfactory network after ON transection. Calcium indicator mixture consisted of 1000 μM Fluo-4/AM (Molecular Probes, Thermo Fisher Scientific), 600 μM MK571 (Sigma-Aldrich), 18 μM Cascade Blue (10,000 MW dextran; Molecular Probes, Thermo Fisher Scientific) in bath solution and the supernatant dye solution was collected after a centrifugation step (1 min, 16.1 rcf). A whole mount preparation was made from a tissue block containing the olfactory system (as described above). A micropipette (Warner Instruments, resistance 10–15 MΩ) filled with dye solution was penetrated into the ventro-lateral part of the OB using a micromanipulator. Dye solution was pressure-injected into the MTC layer at up to three different locations under visual control using epifluorescent illumination. After 35 min of incubation, calcium responses of postsynaptic MTCs in an OB volume were recorded using an upright multiphoton microscope (A1R-MP, Nikon, excitation wavelength: 800 nm). Via fast volumetric resonant scanning, we measured time series of cubic volumes of the amino acid-sensitive, ventrolateral OB (lateral dimensions: 170 μm, 512 × 512 pixel; axial dimension: 120–180 μm; step size: 3–5 μm) at 0.5–1 Hz per image stack.

Preparations were stabilized using a stringed platinum grid in a recording chamber, which was constantly perfused with bath solution applied by gravity feed from a storage syringe through an applicator placed directly above the MOE. The stimuli were applied without stopping the flow and bath solution was constantly removed from the recording chamber through a syringe needle positioned caudally to the preparation. Before application of stimuli, baseline fluorescence was recorded for at least 10 s. After stimulation, fluorescence changes were monitored for at least 50 s. The reproducibility of the responses was verified by regularly repeating the application at least twice. The minimum interstimulus interval was at least 2 min in all of the experiments. All experiments were conducted at room temperature.

### Image and Data Processing

The brightness and contrast of some image stacks from structural measurements were adjusted in the image processing software Fiji (Schindelin et al., 2012)^1^. Spectral imaging and linear unmixing (Zen software, Zeiss) were used to separate overlapping fluorescent signals in slices with active caspase-3 labeling. To quantify cell death and proliferation, active caspase-3-positive and BrdU-positive cells were manually counted. 3D image stacks were visualized in the image processing software Fiji to utilize the volumetric information for the quantification. The average diameter of counted cell profiles was 9 μm, and labeled structures with a diameter smaller than 4 μm or without identifiable nucleus (stained with propidium iodide in active caspase-3 labeled slices) were omitted from the quantification. Multiple sections of each OB were used for quantification. In olfactory organ slices, a randomly chosen 5 μm thick partial sub-volume of the whole acquired image stack was manually quantified.

For the quantification of OB volume, multiple image stacks were stitched together using image processing software Fiji (Preibisch et al., 2009). Cross sectional areas of the transected and non-transected side of the OB were measured at five different levels on the z-axis of the image stack. The sum of these areas was used as an estimate of the OB volume, and the relative change of OB volume between the transected side and the non-transected side was determined for each animal. These changes were calculated for animals killed at different time-points after transection. The relative difference in volume of the left and right hemibulbs of non-transected animals was used as control.

To identify changes in dendritic morphology of individual MTCs after ON transection, a region of interest was cropped out and digitally isolated by restricting the field of view in x-y axis to a single tuft and by making a sub-stack to include only planes containing the tuft. As a last pre-processing step, the image stacks were rendered binary using Otsu’s thresholding method implemented in Fiji (Otsu, 1979). Blunt dendritic terminals were not used in statistical analysis. Tuft morphology was examined using Sholl’s technique for quantitative analysis of complex dendritic branching structures in Fiji (Sholl, 1953; Ferreira et al., 2014). The starting point was centered on the main apical dendrite of MTCs at ~40 μm from the tuft. From there, the dendritic intersections for concentric 3D spheres with stepwise increasing radii of 1 μm were calculated. For the calculation of the average linear tuft-complexity curves, the maxima of intersections of the single tufts of each group were aligned and the curves averaged at each radius of the Sholl analysis. Data visualization and statistical analyses were conducted using custom written Matlab scripts (Mathworks, Natwick).

Changes in calcium indicator fluorescence are given as ∆F/F values in percent. The values were calculated for each pixel according to the following equation: ∆F/F = (F−F_0_)/F_0_. F_0_ represents an averaged pixel fluorescence value derived from the time interval prior to the stimulus and F is the actual pixel fluorescence value at each recorded time point. A response was assumed if the following criteria were met: (i) the maximum amplitude of the calcium transient had to be higher than the maximum of the pre-stimulus intensities; (ii) the onset of the response had to be within ten frames after stimulus application. To facilitate selection of responsive regions of interest, a “pixel correlation map” was obtained by calculating the cross-correlation between the fluorescence signals of a pixel to that of its immediate neighbors (Junek et al., 2009). Analyzed regions of interest in each olfactory epithelium were counted and the number of cells responsive to each stimulus was calculated for each time point. For OB measurements, difference images were calculated for each recorded plane by averaging the fluorescence value of the interval prior to stimulation, and subtracting this value from the mean peak response of the odor induced fluorescence peak (2–3 points for mean peak calculation). Functional imaging data were analyzed using custom written programs in Matlab.

Averaged data are presented as mean ± standard deviation. Statistical significance was determined by Kruskal-Wallis rank sum test followed by Dunn’s multiple comparison *post hoc* test. To control familywise error rate for multiple comparisons a Holm-Bonferroni correction was applied.

### Solutions

Standard bath solution consisted of (in millimolar): 98 NaCl, 2 KCl, 1 CaCl_2_, 2 MgCl_2_, 5 glucose, 5 Na-pyruvate, 10 hydroxyethyl piperazineethanesulfonic (HEPES), 230 mOsmol/l, pH 7.8. High K^+^ bath solution consisted of (in millimolar): 17 NaCl, 80 KCl, 1 CaCl_2_, 2 MgCl_2_, 5 glucose, 5 Na-pyruvate, 10 HEPES, 230 mOsmol/l, and pH 7.8. Adenosine-5′-triphosphate (ATP), 2-methylthio-ATP (2-MeSATP), and bath solution chemicals were purchased from Sigma-Aldrich. Amino acids and purinergic receptor agonists were dissolved in bath solution (10 mM stock) and applied at a final concentration of 100 μM, either individually or as a component in a mixture. All pharmacologic agents were dissolved as concentrated stock solutions, aliquoted and frozen. Aliquots were thawed only once and the working solutions (see specific experiments) were freshly prepared before performing each experiment.

## Results

### Structural and Functional Consequences after Olfactory Nerve Transection in the Main Olfactory Epithelium

To observe structural changes occurring in the MOE after bilateral ON transection, we visualized major cell populations of the MOE, namely supporting cells (SCs), proliferative basal cells (BCs) and ORNs.

**Figure 2:**
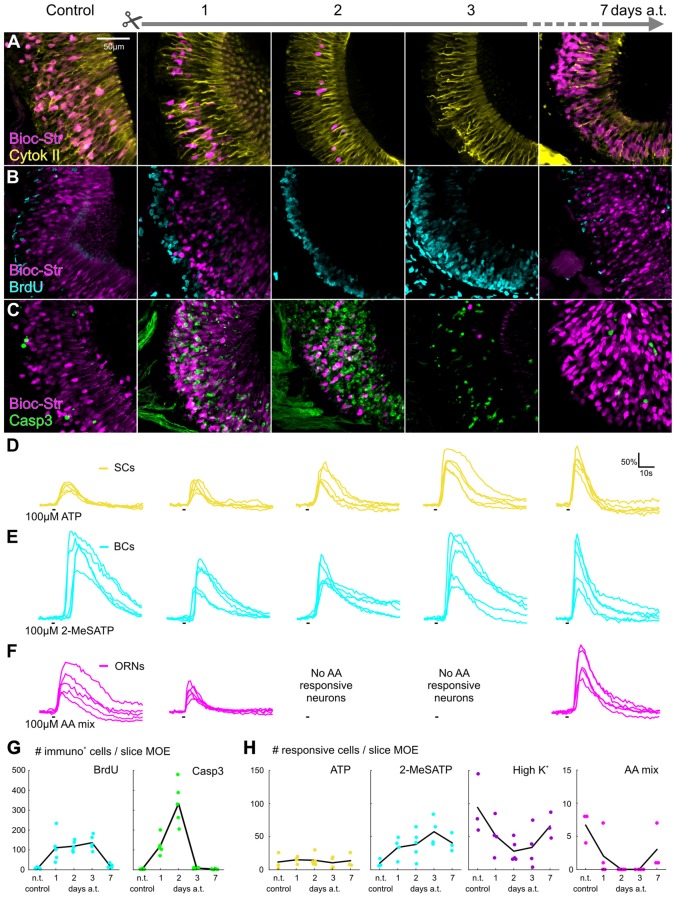
Timeline of structural and functional changes in the MOE after olfactory nerve transection. **(A–C)** Maximum projections of image stacks from representative slices of the MOE before and after ON transection (1, 2, 3 and 7 days). ORNs (Bioc-Str, magenta), Supporting cells (SCs; **A**, yellow), dividing cells (**B**, cyan), apoptotic cells (**C**, green) were labeled and investigated for structural changes post-transection. **(D–F)** Representative calcium transients of five individual cells of one acute slice preparation after stimulation with adenosine-5′-triphosphate (ATP) (**D**, yellow), 2-MeSATP (**E**, cyan) and an amino acid mixture (**F**, magenta). Depicted are a non-transected control and specimens 1, 2, 3 and 7 days post-transection. **(G)** Graphs depicting changes in the number of BrdU positive cells (filled cyan circles), and active caspase-3 positive cells (filled green circles), per slice of the MOE for each time-point analyzed (black lines connect the mean values for each time-point). **(H)** Graphs depicting changes in the number of responsive cells per acute slice of the MOE for each time-point analyzed (black lines connect the mean values for each time-point): ATP-responsive cells located in the SC layer (yellow filled circles), 2-MeSATP-responsive cells (cyan filled circles) and cells activated by high K^+^ bath solution (purple filled circles) and amino acids (magenta filled circles). AA, amino acid; a.t., after transection; BC, basal cell; Bioc-Str, Biocytin-Strepavidin; BrdU, 5-bromo-2′-deoxyuridine; Casp3, active-Caspase3; Cytok II, Cytokeratin type II; MOE, main olfactory epithelium; n.t., non-transected; ON, olfactory nerve; ORN, olfactory receptor neuron; SC, supporting cell.

We found no changes in the morphological structure of the SC layer of the MOE post-transection in comparison to non-transected animals. The apical layer of the MOE exhibited typical cytokeratin II-like immunoreactivity and labeled SCs were clearly visible in control animals, as well as in all analyzed time points post-transection (Figure 2A). Cytokeratin II-positive cells always formed an undisrupted, tightly packed columnar monolayer on the apical surface of the MOE, and spanned their basal processes across the entire epithelium terminating in endfeet-like structures (Hassenklöver et al., 2008). To assess the proliferative activity in the MOE, we conducted BrdU-labeling experiments to visualize cells in the S-phase of mitosis. In healthy control animals, 8 ± 4 BrdU positive cells (*n* = 5) were visible in the BC layer of the MOE, restricted mainly to the area adjacent to the basal lamina (Figures 2B,G). The number of BrdU-positive cells in the MOE was significantly increased 1, 2 and 3 days after transection to 111 ± 76 (*p* = 0.044, *n* = 5), 119 ± 27 (*p* = 0.025, *n* = 5) and 136 ± 37 (*p* = 0.0054, *n* = 5), respectively (Figures 2B,G). The area occupied by proliferating cells was expanded more deeply into the MOE and several labeled cells were also found in intermediate and apical layers (Figure 2B). One week after injury, proliferative activity returned to a level indistinguishable from healthy controls with 19 ± 10 BrdU-positive cells (*n* = 5). To assess the extent of apoptosis in the MOE, we investigated immunoreactivity to a protein involved in programmed cell death, active caspase-3. In the control situation, 4 ± 2 active caspase-3 positive cells (*n* = 5) were visible in the MOE (Figures 2C,G), showing a constricted, bleb-like appearance typical for apoptotic cells (Dittrich et al., 2016). The number of active caspase-3 positive cells was enormously increased 1 and 2 days after ON transection, with 122 ± 48 (*n* = 5) and 333 ± 107 (*p* = 0.0044, *n* = 5), respectively (Figures 2C,G). Labeled cells were found in all layers of the MOE, but the majority was located in the intermediate ORN layer. Three and 7 days post-transection, the number of apoptotic cells decreased back to control levels with 8 ± 3 (*n* = 5), and 3 ± 2 (*n* = 5) labeled cells, respectively (Figures 2C,G). The population of ORNs, labeled via the ON during transection, was severely affected with rapidly decreasing numbers of ORNs during the first 2 days, and virtually none 3 days post-transection (Figures 2A–C).

We analyzed functional changes in the various cell populations of the MOE after ON transection. As stimulations, we applied a solution containing either a purinergic receptor agonist, ATP or 2-MeSATP, to induce responses in non-neuronal cells or a mixture of amino acids to activate amino acid-sensitive ORNs. ATP has been shown to activate cells in both the SC and BC layers, whereas 2-MeSATP activates only cells in the BC layer, thus allowing us to make a distinction between the two cell populations based on the different response profiles (Hassenklöver et al., 2008, 2009). A high K^+^ bath solution was used to generally activate all ORNs. All stimulations were applied to every slice preparation. A total of 19 slices that included the lateral portion of the OE, and that exhibited responsive cells were used in statistical analysis. Slices were obtained from non-transected control animals (*n* = 3) and from animals killed 1, 2, 3 and 7 days post-transection (*n* = 4, 5, 4 and 3, respectively). We found that not only were ATP and 2-MeSATP responsive cells still present in the SC and BC layer of the MOE after nerve transection, but the number of responsive cells was increased in the BC layer, where the proliferative stem cell population resides (Figures 2D,E,H). The average number of cells per slice with stable responses exclusively to ATP, located in the SC layer, showed no significant increase at any of the time-points observed when compared to control (12 ± 13). No significant difference from the control situation was observed 1, 2, 3 or 7 days after ON transection (15 ± 5; 14 ± 9; 11 ± 9; 14 ± 11; Figure 2H). All slices analyzed showed cells responsive to ATP. Representative example traces of ATP responsive cells are depicted in Figure 2D (five cells from one MOE slice per time-point). The average number of cells per slice with stable responses to 2-MeSATP showed a slight increase from 10 ± 6 in the control situation to 34 ± 16, 38 ± 21, 58 ± 21 and 41 ± 14, 1,2,3 and 7 days after transection, respectively (Figure 2H). All slices analyzed showed cells responsive to 2-MeSATP, and responsive cells were found to be located in the BC layer of the MOE. Example traces of 2-MeSATP responsive cells can be seen in Figure 2E (five cells from one MOE slice per time-point). We found that the average number of high K^+^ responsive cells in the MOE decreased progressively from 94 ± 45 in the control situation, to 51 ± 27 1 day after transection, and 28 ± 16 2 days after transection (Figure 2H). It began to increase 3 days after nerve transection (34 ± 31), and was progressively higher 1 week after transection (66 ± 20). To further analyze functional changes in the ORN population we applied 100 μM amino acid mix, natural water born odorants indicative of the presence of food in the environment, to the MOE. A sub-population of ORNs has been shown to respond to amino acids and has been found to be predominantly located in the lateral portion of the MOE (Gliem et al., 2013). In the normal MOE, 7 ± 2 ORNs were responsive to the amino acid mixture. No significant difference in the number of amino acid responsive cells was found 1 day post-transection (2 ± 3). We found no amino acid responsive cells in the MOE 2, and 3 days post-transection (Figures 2F,H), a significant decrease from the control situation (*p* = 0.021 and *p* = 0.029, respectively). Responses were again observable 1 week after nerve transection (3 ± 3), showing no significant difference when compared to the control situation. Example traces of amino acid responsive ORNs can be seen in Figure 2F (five cells from one MOE slice per time-point).

### Relative Changes in Olfactory Bulb Volume—Axon Degradation and Reinnervation by Newly Formed Olfactory Sensory Neurons

**Figure 3:**
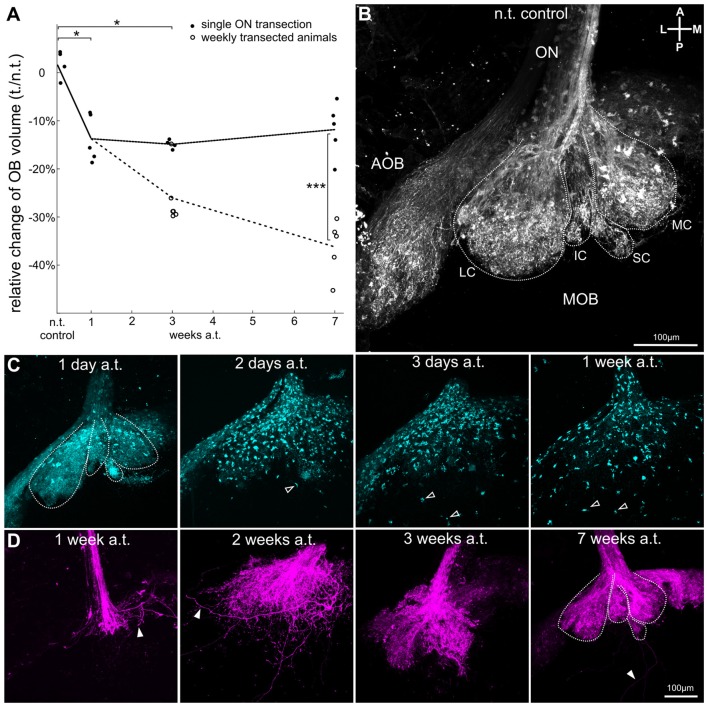
Olfactory nerve transection induces transitional OB volume reduction due to axonal degradation of olfactory receptor neurons and subsequent reinnervation by new neurons. **(A)** Graph shows relative changes in OB volume recovering after ON transection (filled black circles, black line connects mean values for each time-point analyzed) and of animals subjected to weekly ON transection to hinder reconnection of ORN axons to the OB (empty circles, dotted line connects mean values for each time-point analyzed). Animals were transected unilaterally, and changes in OB volume are shown as the percentage of decrease in volume of the transected side in relation to the non-transected side. **(B)** Non-transected OB with ORN axons (white) stained by nasal electroporation of fluorescent dextrans. Typical ventral glomerular clusters are outlined with a dotted white line: lateral (LC), intermediate (IC), small cluster (SC) and medial cluster (MC). The ORN axons of the accessory olfactory bulb (AOB) are also visible on the lateral side of the OB. **(C)** ON transection induces gradual axonal degradation in the OB. Axons (cyan) were labeled by microRuby via the ON, which is anterogradely transported along the axons. Two days post-transection degeneration of axonal fibers became apparent and fluorescent dye began to accumulate in aggregates that gradually dispersed through the OB over time (posterior agglomerates highlighted by open arrowheads, glomerular clusters are outlined with a dotted white line). **(D)** Representative images of the OB showing reconnecting ORN axons (magenta) stained by nasal electroporation at different time-points after ON transection (1, 2, 3 and 7 weeks). Examples of individual axons are highlighted by filled arrowheads and glomerular clusters are outlined with a dotted white line. A, anterior; AOB, accessory olfactory bulb; a.t., after transection; IC, intermediate cluster; L, lateral; LC, lateral cluster; M, medial; MC, medial cluster; MOB, main olfactory bulb; n.t., non-transected; OB, olfactory bulb; ON, olfactory nerve; ORN, olfactory receptor neuron; P, posterior; SC, small cluster. Statistical significance was tested using Kruskal-Wallis test followed by Dunn’s multiple comparison *post hoc* test with Holm-Bonferroni correction (*p < 0.05, ***p < 0.001).

Experiments performed on the olfactory organ (described above) have shown that extensive cell death occurs in the population of ORNs in the first days following ON transection. We compared the relative change in volume between the hemibulb on the transected side with that on the non-transected side for each animal killed at different timepoints after transection. The hemibulbs of non-transected control animals were almost of identical volume (2 ± 3%, *n* = 4). We found that the hemibulb on the transected side decreased significantly by 13 ± 6% in relation to the non-transected side within 1 week post-transection (*p* = 0.048, *n* = 5), and was still decreased after 3 weeks with a volume reduction of 15 ± 1% (*p* = 0.048, *n* = 4, Figure 3A, filled circles). Seven weeks after ON transection, the hemibulb on the lesioned side showed a tendency towards recovery and the volume was only 11 ± 6% (*n* = 5) smaller than the intact control side. We hypothesize that the observed OB volume changes are due to the loss of axonal input and subsequent reinnervation by axons of newly generated ORNs during recovery. Weekly ON transections effectively impedes ORNs from reconnecting to the OB, and allows us to observe how the OB is affected by this lack of input over a longer period. Repeatedly transected animals showed a significant decrease in OB volume of 25 ± 7% after 3 weeks (*p* = 0.015, *n* = 6) and of 36 ± 7% 7 weeks after initial ON transection (*p* = 0.00027, *n* = 5). The decrease in OB volume was significantly larger 7 weeks after weekly transection in comparison to recovering animals (*p* = 0.027, Figure 3A, open circles).

To observe the cause of these volume reductions after ON transection, we investigated the fate of injured receptor neurons in the OB and their axonal degeneration. The axonal projection of ORNs into the OB in healthy *Xenopus laevis* larvae is depicted in Figure 3B highlighting the characteristic organization into lateral, intermediate (including small cluster), and medial glomerular cluster. We found that the onset of axon fragmentation does not begin until after the first day post-transection, as seen in the first left hand panel of Figure 3C, where the glomerular clusters are still clearly discernible and the ON is mostly intact (*n* = 5). Two days after transection we found that the axon terminals and clusters start to disassemble and the dye used to label the ORNs accumulated throughout the glomerular layer (open arrowheads, *n* = 6). Three days post-transection most axon fibers were degraded, dye accumulations were more dispersed across the OB, and were eventually found in more caudal layers of the OB, towards the ventricular system (*n* = 5). One week post-transection none of the original axon fibers or cluster organization was identifiable anymore and the dye from former axonal staining was highly dispersed (*n* = 7).

Our experiments performed on the olfactory organ have shown that already 1 week post-transection functional, odorant-sensitive ORNs reappear in the olfactory epithelium (Figure 2). This indicates that also in this time window a reinnervation of the OB could potentially occur. To verify this hypothesis, we stained ORNs to determine when axons of newly formed ORNs reach the OB and begin the process of reinnervation (Figure 3D). We found that already 1 week post-transection some newly formed ORN axons reach the OB via the reestablished ON (*n* = 6). However, the axon terminals were restricted to a small area close to the ON and did not show glomerular arborizations. After 2 weeks, the number of labeled axons and the covered area in the OB was increased (*n* = 4). Nevertheless, many axons exhibited long branches without being correctly connected and a proper organization into glomerular clusters was still missing (Figure 3D, filled arrowheads). Reinnervation progressed in the following weeks as pioneering axons began to form organized structures resembling normal glomerular clustering around 3 weeks after ON transection (*n* = 6). Although the progress of reinnervation varied between animals, at 7 weeks post-transection most OBs exhibited a glomerular cluster organization similar to the non-transected controls (*n* = 5).

### Increased Cell Death in the Olfactory Bulb Following Olfactory Nerve Transection

**Figure 4:**
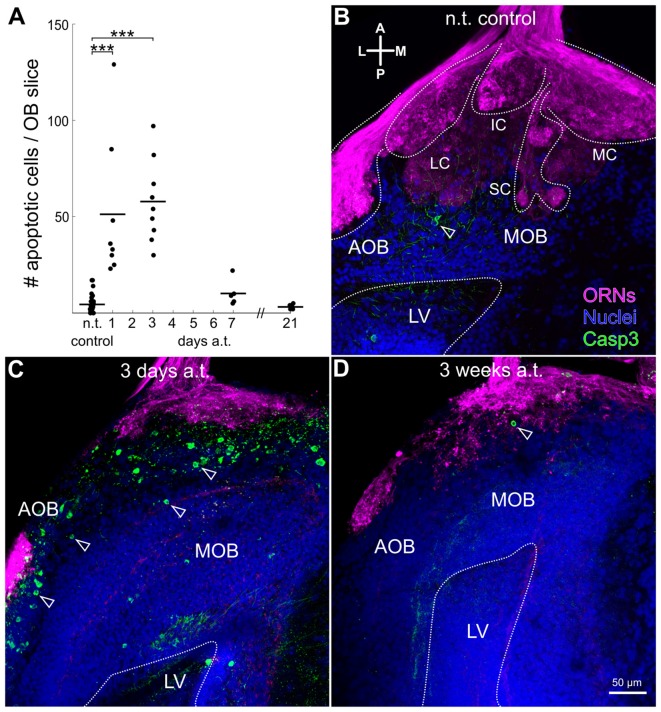
Increased levels of apoptotic cells in anterior layers of the OB after olfactory nerve transection. **(A)** Graph depicts changes in the number of apoptotic cells in slices of the OB at different time-points over the course of 3 weeks after ON transection. Maximum projection images of representative slices of the OB of a non-transected control animal **(B)**, an animal killed 3 days post-transection **(C)** and 3 weeks post-transection **(D)**, with biocytin-streptavidin stained ORNs (magenta), active caspase-3 staining of apoptotic cells (green), and propidium-iodide staining of all cell nuclei (blue). Distinct glomerular clusters and lateral ventricle are outlined with dotted white lines. Open arrow heads highlight cell bodies undergoing apoptosis. A, anterior; AOB, accessory olfactory bulb; a.t., after transection; Casp3, active-Caspase3; IC, intermediate cluster; L, lateral; LC, lateral cluster; LV, lateral ventricle; M, medial; MC, medial cluster; MOB, main olfactory bulb; n.t., non-transected; OB, olfactory bulb; ON, olfactory nerve; ORN, olfactory receptor neuron; P, posterior; SC, small cluster. Statistical significance was tested using Kruskal-Wallis test followed by Dunn’s multiple comparison *post hoc* test with Holm-Bonferroni correction (***p < 0.001).

Being that ON transection had a clear impact on the OB volume, we investigated if postsynaptic neurons of the OB network were also affected as a next step. To assess potential cell loss in the OB we performed active caspase-3 immunostainings on slices of the OB. We found that caspase-3 mediated cell death also occurred in cells located in the OB rapidly after ON transection (Figure 4). Apoptotic cells were located predominantly in the ON layer and in the glomerular layer of the OB (Figure 4C). The number of apoptotic cells counted in each slice of the OB increased significantly from 5 ± 5 in the control situation (*n* = 25), to 51 ± 37 just 1 day after ON transection (*p* = 0.00034, *n* = 8) and to 58 ± 22 3 days post-transection (*p* = 0.000016, *n* = 9; Figure 4A). After 1 and 3 weeks there is no significant difference when compared to the control situation with 10 ± 7 (*n* = 5), and 3 ± 1 (*n* = 7) labeled apoptotic cells, respectively (Figures 4A,B,D).

### Dynamic Changes of Dendritic Tuft Complexity in Mitral/Tufted Cells Following Olfactory Nerve Transection

**Figure 5:**
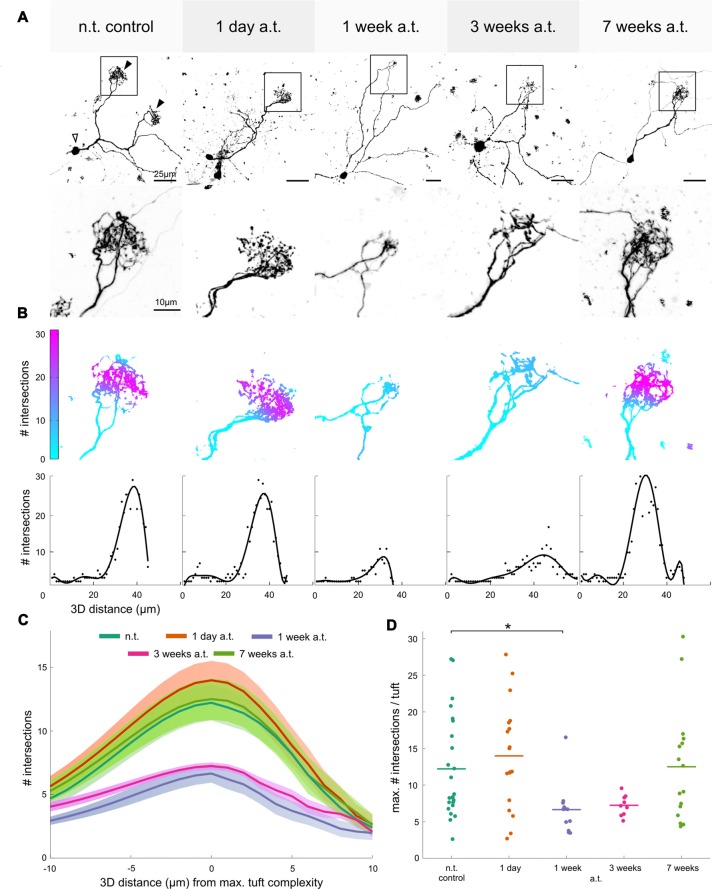
Dynamic changes of mitral/tufted cell dendritic tuft complexity in the OB after olfactory nerve transection. **(A)** Top row shows individual MTCs stained via sparse cell electroporation. Maximum intensity projections of image stacks of representative MTCs are shown for each time-point after ON transection. Animals were transected unilaterally and MTCs were stained and analyzed on both the non-transected side of the OB, used as control, and on the transected side, 1, 3 and 7 weeks a.t. Bottom row shows a magnification of the tufted regions (boxed outline). **(B)** Top row illustrates quantification of complexity of the tufts shown in **(A)** using Sholl analysis. The number of intersections on the three-dimensional tuft is represented as a color gradient on the tuft morphology—blue areas indicate very few intersections and magenta indicates many intersections. Bottom row shows linear Sholl plots for each of the presented tufts with number of intersections indicated as dots and best fit polynomial function as line. **(C)** The average linear tuft-complexity curves (of all curves as shown in **B**) for tufts of each respective group are shown. A distance of ±10 μm around the maximum is shown. The shaded areas around the curves indicate the SEM within each group. **(D)** Scatter plot showing the maximum number of intersections for each tuft analyzed in the control group and at each respective time point a.t. Lines show the mean of all analyzed tufts for each time-point. a.t., after transection; MTC, mitral/tufted cell; n.t., non-transected; OB, olfactory bulb; ON, olfactory nerve. Statistical significance was tested using Kruskal-Wallis test followed by Dunn’s multiple comparison *post hoc* test with Holm-Bonferroni correction (*p < 0.05).

As cell death has mostly been observed in the glomerular layer, it imposes the question whether the MTCs located in the deeper OB layers also suffer from consequences related to denervation. In Figure 5A different, individual MTCs from ON-transected animals at different time intervals after lesion are presented in comparison to a non-transected control. In *Xenopus*, MTC dendrites have been found to bifurcate and innervate multiple glomeruli (see also Nezlin and Schild, 2000). These dendrites end in fine arborizations full of dendritic varicosities, called dendritic tufts (highlighted by squares in Figure 5A). Dendritic tufts of MTCs that form the postsynaptic component of a glomerular module were clearly smaller and less complex 1 week post-transection. Complexity of dendritic tufts was quantified using Sholl analysis as depicted in Figure 5B. For the representative MTC tufts shown, the number of intersections in general is clearly reduced after 1, and 3 weeks following ON transection (Figure 5B). Very complex tufts exhibited a maximum number of intersections well above 20, while minimally complex tufts featured a maximum below 10 intersections (Figures 5B–D). In control OBs, a mean maximum number of 12.2 ± 7.0 intersections was measured (*n* = 24 tufts from 12 animals), which was not significantly different from 14.0 ± 7.2 intersections 1 day after transection (*n* = 18 tufts from 13 animals, Figure 5D). After the loss of presynaptic input, these structures show a gradual decrease in complexity and tufts were significantly less complex 1 week after transection), with an average maximum of 6.6 ± 3.5 intersections per tuft (*p* = 0.039, *n* = 12 tufts from 9 animals, Figures 5C,D). Three weeks after transection and 7 weeks after transection the mean maximum number of intersections was 7.2 ± 1.4 (*n* = 9 tufts from 7 animals) and 12.5 ± 7.7 (*n* = 16 tufts from 8 animals), respectively, no longer significantly different from control. This suggests a tendency for the tufts to become more complex after ORNs have begun to reconnect to the OB (see above). The lowered MTC tuft complexity after 1 week is also visible in the mean linear Sholl plots of the region around the maximum number of intersections (Figure 5C).

These results indicate that MTC tufts show a highly plastic response to the loss of their presynaptic partner. Dendritic tuft complexity reached its minimum value right before reconnection of ORN axons to the bulb started and returned to normal morphology after 7 weeks, when glomerular clusters recovered their structure and again resembled the control situation.

### Recovery of Odorant-Induced Responses in the Glomerular Layer and Mitral/Tufted Cells of the Olfactory Bulb

**Figure 6:**
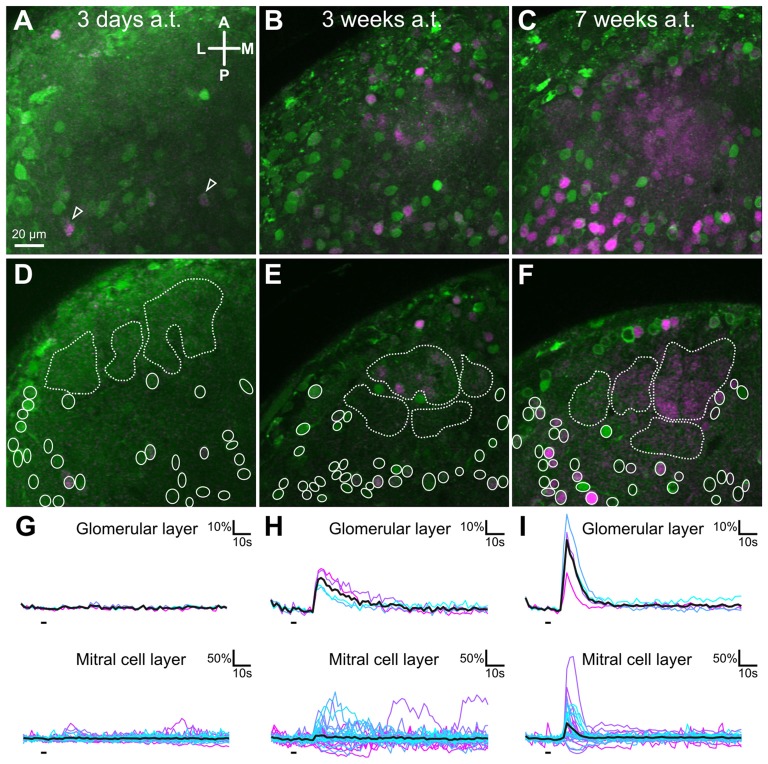
Functional changes in mitral/tufted cell and glomerular layer of the lateral glomerular cluster of the OB after olfactory nerve transection. Maximum projections of representative examples of imaged volumes in the ventro-lateral OB of different whole olfactory system explants measured 3 days **(A)**, 3 weeks **(B)** or 7 weeks **(C)** post-transection. MTC somata and their tufts were labeled by calcium indicator injection (green) and responses to odorant stimulation of the olfactory organ were recorded. Regions that showed a time-correlated response to stimulation of the MOE with an amino acid mixture are shown in magenta (Difference image of peak response minus pre-stimulus activity). Single planes of the imaged volumes measured 3 days **(D)**, 3 weeks **(E)** or 7 weeks **(F)** after ON transection. Dashed white lines surround regions of interest in the glomerular layer, while white ellipses indicate MTC somata. **(G–I)** Calcium transients of neuropil and individual cells (different shades of blue and magenta, shown as ∆F/F values) are derived from the regions of interests highlighted in the respective images above. The mean response of all regions of interest in the glomerular neuropil and mitral cell layer are depicted as black traces. Some cells with occasional, spontaneous, time-correlated activity were visible (also highlighted with open arrowheads). A, anterior; a.t., after transection; L, lateral; M, medial; MOE, main olfactory epithelium; MTC, mitral/tufted cell; OB, olfactory bulb; ON, olfactory nerve; P, posterior.

We performed multi-photon calcium imaging of MTCs to investigate the extent of functional recovery of the olfactory network after ON transection. Calcium responses of the lateral MTC population’s tufts and somata were recorded after application of amino acids to the olfactory epithelium. In animals measured 3 days after transection no odor induced activity could be detected on the postsynaptic level, neither in MTC glomerular tufts nor in MTC somata (Figures 6A,D,G; *n* = 3). Only very rarely, somatic calcium signal increases occurred time-correlated with odor stimulus application (Figure 6A, arrowheads). Given the high spontaneous activity we observed among the MTCs at this time point as well as the lack of any glomerular responses, it can be assumed that those calcium events were coincidentally time-correlated to the stimulus (Figure 6G). In animals measured 3 weeks after transection, glomerular as well as somatic responses to amino acid stimulation were present (Figures 6B,E; *n* = 3). Even though the extent of reactive glomerular neuropil and MTC somata varied among the three animals recorded for this time point, odorant induced signals could be detected in each preparation in both the glomerular and MTC layer (Figure 6H). In animals that were measured after 7 weeks post-transection, large portions of the lateral MTC population and the lateral glomerular array were activated by the amino acid stimuli (Figures 6C,F; *n* = 5). Characteristic odorant induced calcium transients were much more distinctly observable in both layers of the OB (Figure 6I) and the glomeruli showed individual response profiles to five different, individual amino acids applied (data not shown). In summary, ON transection led to disruption of amino acid induced MTC activity, but the functional OB circuitry was gradually restored over the time course of 7 weeks.

## Discussion

Post-developmental neurogenesis is restricted to only a small number of areas in the nervous system. The telencephalic subventricular zone and the peripheral olfactory epithelium are two areas of special interest, as they provide new functional elements to both sides of the fundamental olfactory circuit. On the peripheral side, multipotent stems cells of the BC layer of the olfactory epithelium replenish its different cellular components (Graziadei and Graziadei, 1979; Huard et al., 1998; Leung et al., 2007). ORNs regularly die and have to be replaced with new sensory neurons that reintegrate into a functioning circuit in the brain (Farbman, 1990; Roskams et al., 1996; Schwob, 2002). Beyond that, neurogenesis in the olfactory epithelium can also occur on a larger scale following injury (Schwob, 2002). Severing the ONs of larval *Xenopus laevis* allowed us to successfully eliminate olfactory input to the OB and consequently cause the loss of olfactory function. We show the progression of events that occur after lesion and how the individual components of the olfactory network are transiently recovering, both structurally and functionally.

### Timeline for Regeneration in the Olfactory System of Larval *Xenopus laevis* after Olfactory Nerve Transection

Degeneration in the olfactory epithelium is immediately apparent within the first 3 days following ON transection. We observed a pronounced increase in apoptotic cells throughout the olfactory epithelium, leading to neuronal loss. In particular, a decrease in the overall number of ORNs activated by high K^+^ bath solution and a complete loss of amino acid-responsive ORNs was detected. The overall number of excitable ORNs decreased considerably after transection, but these cells were at no time completely eradicated from the epithelium. This is most likely due to a remaining population of immature ORNs, which have not yet established an axonal connection to the OB and thus are unaffected by ON transection (Schwob, 2002).

The morphology of the SC layer was unaltered by ON transection, and the number of cells responsive to purinergic receptor agonists in the SC and BC layers did not decrease at any moment surveyed, indicating that the induced cell death in the olfactory epithelium is restricted mainly to the neuronal population. It has been shown that purinergic signaling pathways influence cell turnover in the healthy MOE (Hassenklöver et al., 2009; Jia et al., 2009), and could potentially play an important role in regulating neuroproliferation during the process of regeneration after lesion (Jia and Hegg, 2010; Jia et al., 2013). After ON transection we found an increase in the number of cells responsive to the application of 2-MeSATP, shown to activate exclusively cells in the BC layer, where the population of stem and progenitor cells of the olfactory epithelium reside (Schwob, 2002; Hassenklöver et al., 2009). This change presumably reflects upregulated proliferative activity in response to lesion and in succession increasing numbers of 2-MeSATP-sensitive progenitor cells. In fact, following injury the stem cell population responds almost immediately to increased neuronal death with a significant increase of BrdU-positive, proliferating cells.

Truncated axon terminals of ORNs were subsequently found to be degenerating and no odor-induced activity could be detected on the postsynaptic level in the OB indicating immediate loss of olfactory function following lesion. In the OB, we also saw a significant increase in caspase-mediated cell death in the first 3 days immediately following ON transection. Apoptotic cell somata were restricted mainly to the ON layer and the glomerular layer of the OB, suggesting dying periglomerular cells (Nezlin and Schild, 2000). This is also supported by our recording of spontaneously active neurons in the MTC layer of the OB and our successful labeling of individual MTCs. Thus, we conclude that MTC are not the main cell population of the OB affected by cell death.

During the process of regeneration stem cell proliferation in the MOE initiates a compensatory increase in immature neurons expressing NCAM-180 (Cervino et al., 2017). Eventually, this leads to an elevation of functional ORNs over the course of 1 week after ON transection (this study). This is similar to newborn ORNs in the rodent MOE that are mature after 7–8 days expressing olfactory marker protein (Miragall and Graziadei, 1982). In *Xenopus*, recovery of OMP-expression in newly generated ORNs takes longer than 7 days and remains reduced even 4 weeks post-transection (Cervino et al., 2017). Already after 1 week, other surveyed parameters in the *Xenopus* MOE, e.g., functional odorant responses, return to control levels. This period is comparable to the recovery time of the *Xenopus* MOE after chemical lesion that leads to widespread cell death in multiple cell populations, including SCs and ORNs (Frontera et al., 2016).

The network of the OB is still functionally disrupted 1 week post-transection, however, initial signs of a recovering OB are observable as first pioneering ORN axons project into the OB. Axonal reinnervation occurs progressively over the next weeks and already 3 weeks after transection the glomerular cluster organization is coarsely restored. At this point the first odor-evoked responses reappear in the glomerular neuropil of the lateral cluster of the OB. Thus, the functional integration of newborn ORNs into the OB circuitry takes much longer than the structural recovery of the olfactory epithelium. Seven weeks after transection, ORN axons in the OB have reformed glomerular clusters structurally similar to those found in healthy non-transected animals and amino acid-induced responses in the neuropil of the lateral glomerular cluster are comparable to healthy control animals. Nevertheless, it cannot be excluded that some amount of miswiring occurs during recovery after injury. In our study, the structural and functional recovery of the olfactory system of *Xenopus* *laevis* larvae after ON transection took 7 weeks. A recovery of specific foraging behavior in response to food-odor occurs 3 weeks after ON transection (Cervino et al., 2017). Notably, newly formed ORN synapses in larval *Xenopus* show a high vesicle density in their active zones and can be active before the glomerular units of the OB are fully restored (Terni et al., 2017). In experiments performed on rodents, the average time of recovery is approximately 2.5 months for both ON transection and chemical lesion, notwithstanding differences in the extent of recovery (Kobayashi and Costanzo, 2009; Blanco-Hernández et al., 2012; Cheung et al., 2014; Murai et al., 2016).

### Olfactory Map Recovery and Injury-Induced Dendritic Reshaping of Second-Order Neurons

A variety of factors influence the efficacy of olfactory system restoration after injury, and in many cases, the recovery of olfactory network functionality is only partial. When the ONs and OBs are injured, newly regenerated ORN axons must find their way back to their appropriate OB location and overcome challenging obstacles, like scar tissue formation and gliosis, in order to preserve the spatial mapping of odorants (Kobayashi and Costanzo, 2009). Different from developmental OB network formation, targeting of ORN axons to glomeruli is erroneous in many cases after successful recovery of the olfactory system (Blanco-Hernández et al., 2012; Cheung et al., 2014; Murai et al., 2016). Axonal pathfinding during developmental olfactory map formation is governed by olfactory receptor gene identity, ORN cell type, molecular guidance cues, and activity-dependent mechanisms (for review see Nishizumi and Sakano, 2015). At least to some extent these mechanisms are also active in the adult organism (Blanco-Hernández et al., 2012; Cheung et al., 2014). It has been suggested that regenerating ORNs depend more on axon-axon interactions in the adult (Imai and Sakano, 2011), making recovery of the olfactory map after ON transection difficult. Consistent with this idea, chemical lesions to the olfactory epithelium, in which ORNs are not entirely eliminated, do not lead to map distortions that are as severe (Blanco-Hernández et al., 2012).

The postsynaptic side of the OB also plays an important role in the formation and recovery of the olfactory map. Dendrites are dynamic structures whose main function consists of integrating synaptic input, a process on which they depend in order to maintain structural integrity (Tavosanis, 2012). This is attributed largely to the delivery of neurotrophins from the pre-synapse that enhance dendritic growth and branching as well as synaptogenesis (Imamura and Greer, 2009). In the OB, the axons of receptor neurons form glutamatergic synapses with dendrites of MTCs and periglomerular cells (Nagayama et al., 2014). In mammals, mitral cell dendrites are reshaping during an early postnatal phase (Meisami and Safari, 1981; Malun and Brunjes, 1996), but remain stable in the adult olfactory system (for review see Mizrahi and Katz, 2003). Elimination of ORN axons during early developmental stages, before formation of mature dendritic tufts, leads to atrophy of mitral cell dendrites (Couper Leo and Brunjes, 2003). In the adult mouse, ON transection initiates dendritic retraction of mature mitral cells and re-establishment of connectivity is reduced even after ORN projection recovery (Murai et al., 2016). In non-mammalian vertebrates, like zebrafish, extensive loss of dendritic branches occurs after ablation of the MOE, transection or chemical damage to ORNs (Byrd, 2000). In our study, we found that the dendritic tufts of MTCs in the OB substantially lose complexity and dendritic varicosities within 1 week after ON transection. The lack of input from the ORN axons appears to have a degenerative effect on the fine branches of the MTCs, which retract and gradually lose their primary synaptic contact site. However, our results show that in the amphibian olfactory system, MTCs retain the capacity to recover their complexity upon reinnervation, and the olfactory system is eventually functionally restored.

Structural integrity of MTC dendrite tufts and the OB in general seems to be dependent on afferent innervation. In *Xenopus*, it has previously been shown that, after removal of the telencephalon, reconnection of the ON is essential for recovery of the OB to occur (Yoshino and Tochinai, 2006). The effect of afferent fibers on their target regions seems crucial to prevent continuous degradation of the OB structure. This has also been demonstrated to be true during development, as a quantitative correlation has been found between the number of ORNs reaching the OB and the number of MTCs in the developing OB of *Xenopus laevis* (Byrd and Burd, 1991, 1993). We showed that when ORNs are not allowed to reconnect to the OB, due to continuous weekly transections of the ON, a progressive decrease in OB volume with no apparent recovery was observed. This decrease in volume was significantly larger from experiments with only a single nerve transection. A reduction in OB volume is also caused by sensory deprivation in other animal models and permanent denervation can reduce OB volume by up to −50% when compared to control (rabbit: Matthews and Powell, 1962; frog: Graziadei and DeHan, 1973; rat: Meisami and Safari, 1981; zebrafish: Byrd, 2000). This is an indication that extensive damage and cell loss is occurring in the OB in consequence of longer periods without afferent innervation. It seems that MTCs have evolved the ability to survive the loss of their presynaptic partner, at least temporarily and to a certain extent.

This is especially relevant in the *Xenopus* olfactory system that undergoes extensive changes during the process of metamorphosis, which occurs shortly after recovery is complete in our experiments. Animals were chosen for this study according to their stage of larval development in order to ensure that the changes that occur in the olfactory system of larval *Xenopus laevis* during metamorphosis would not overlap with our time window of investigation (Dittrich et al., 2016). During metamorphic reorganization of the olfactory organ, whole ORN populations perish, shift locations and a substantial functional rewiring of the system takes place (Dittrich et al., 2016; Syed et al., 2016). Thus during the process of normal development, MTCs of *Xenopus* need to be capable of surviving an extended period with diminished axonal input. This might also be advantageous during recovery after injury.

Maintaining the ability to survive without peripheral input is helpful in a system where continual neurogenesis occurs, but it is still unclear what factors limit the survival of MTCs. It is known that a population of stem cells exists in the subventricular zone that is responsible for the lifelong turnover of interneurons (Kaplan and Hinds, 1977), but post-developmental generation of MTCs and recapitulation of complex wiring to higher brain centers seems impossible. Previous studies have shown that during the process of regeneration after lesion in larval *Xenopus*, there is an increase in BCs expressing BDNF, as well as an increase in the ON and OB (Frontera et al., 2015). In the developing mammalian olfactory system, there is evidence that MTCs express the TrkB neurotrophin receptor, and that BDNF affects dendritic morphology and stimulates branching (Imamura and Greer, 2009). We could speculate that the permissive environment that allows circuit restoration is related in part to the presence of neurotrophic factors. It could be that larval *Xenopus* exhibit elevated neurotrophic support to sustain the viability of MTCs during neuroregeneration and olfactory network reorganization that the adult mammalian system is lacking.

## Conclusion

The larval *Xenopus* olfactory system shows a high degree of resilience to injury and a robust capacity for olfactory network recovery. Over the course of approximately 2 months, the structure and function of the OB degenerates (cell death is increased, axons degrade, glomerular clusters disappear and dendritic tuft complexity of second order neurons is reduced), and subsequently recovers (reinnervation with new ORNs occurs, cluster organization is restored, second order neurons regain complexity and glomerular responses return). Our study forms the basis for further investigations on factors that can influence successful olfactory system recovery after injury. It is not yet known what specific changes occur in the MTC population after the loss of their presynaptic partner. Of special interest could also be to assess the precision of the restored olfactory map and to investigate mechanisms beneficial for recovery of olfactory perception.

## Author Contributions

The experiments were conceived and designed by TH and IM. The experiments were performed by SJH, LW, TO and KD. SJH, LW, TO and TH analyzed the data. SJH, TH and IM wrote the article. All authors participated in the discussion of the data and in production of the final version of the manuscript.

## Conflict of Interest Statement

The authors declare that the research was conducted in the absence of any commercial or financial relationships that could be construed as a potential conflict of interest.

## Footnotes

^1^http://fiji.sc/Fiji

## References

[B1] Blanco-Hernández E. Valle-Leija P. Zomosa-Signoret V. Drucker-Colín R. Vidaltamayo R. (2012). Odor memory stability after reinnervation of the olfactory bulb. PLoS One 7:e46338. 10.1371/journal.pone.0046338 23071557 PMC3468571

[B2] Brann J. H. Firestein S. J. (2014). A lifetime of neurogenesis in the olfactory system. Front. Neurosci. 8:182. 10.3389/fnins.2014.00182 25018692 PMC4071289

[B4] Byrd C. A. (2000). Deafferentation-induced changes in the olfactory bulb of adult zebrafish. Brain Res. 866, 92–100. 10.1016/s0006-8993(00)02252-6 10825484

[B5] Byrd C. A. Burd G. D. (1991). Development of the olfactory bulb in the clawed frog, *Xenopus laevis*: a morphological and quantitative analysis. J. Comp. Neurol. 314, 79–90. 10.1002/cne.903140108 1797876

[B3] Byrd C. A. Burd G. D. (1993). The quantitative relationship between olfactory axons and mitral/tufted cells in developing *Xenopus* with partially deafferented olfactory bulbs. J. Neurobiol. 24, 1229–1242. 10.1002/neu.480240909 8409980

[B6] Caviness V. S. Jr. Takahashi T. Nowakowski R. S. (1995). Numbers, time and neocortical neuronogenesis: a general developmental and evolutionary model. Trends Neurosci. 18, 379–383. 10.1016/0166-2236(95)93933-o 7482802

[B7] Cervino A. S. Paz D. A. Frontera J. L. (2017). Neuronal degeneration and regeneration induced by axotomy in the olfactory epithelium of *Xenopus laevis*. Dev. Neurobiol. 77, 1308–1320. 10.1002/dneu.22513 28719101

[B8] Cheung M. C. Jang W. Schwob J. E. Wachowiak M. (2014). Functional recovery of odor representations in regenerated sensory inputs to the olfactory bulb. Front. Neural Circuits 7:207. 10.3389/fncir.2013.00207 24431990 PMC3882662

[B9] Christie K. J. Turnley A. M. (2013). Regulation of endogenous neural stem/progenitor cells for neural repair-factors that promote neurogenesis and gliogenesis in the normal and damaged brain. Front. Cell. Neurosci. 6:70. 10.3389/fncel.2012.00070 23346046 PMC3548228

[B10] Coelho D. H. Costanzo R. M. (2016). Posttraumatic olfactory dysfunction. Auris Nasus Larynx 43, 137–143. 10.1016/j.anl.2015.08.006 26441369

[B11] Couper Leo J. M. Brunjes P. C. (2003). Neonatal focal denervation of the rat olfactory bulb alters cell structure and survival: a Golgi, Nissl and confocal study. Dev. Brain Res. 140, 277–286. 10.1016/s0165-3806(02)00614-4 12586433

[B12] Cowan C. M. Roskams A. J. (2002). Apoptosis in the mature and developing olfactory neuroepithelium. Microsc. Res. Tech. 58, 204–215. 10.1002/jemt.10150 12203699

[B13] Cummings D. M. Snyder J. S. Brewer M. Cameron H. A. Belluscio L. (2014). Adult neurogenesis is necessary to refine and maintain circuit specificity. J. Neurosci. 34, 13801–13810. 10.1523/JNEUROSCI.2463-14.2014 25297106 PMC4188975

[B14] Dittrich K. Kuttler J. Hassenklöver T. Manzini I. (2016). Metamorphic remodeling of the olfactory organ of the African clawed frog, *Xenopus laevis*. J. Comp. Neurol. 524, 986–998. 10.1002/cne.23887 26294036

[B15] Doty R. L. Yousem D. M. Pham L. Kreshak A. Geckle R. Lee W. (1997). Olfactory dysfunction in patients with head trauma. Arch. Neurol. 54, 1131–1140. 10.1001/archneur.1997.00550210061014 9311357

[B16] Farbman A. I. (1990). Olfactory neurogenesis: genetic or environmental controls? Trends Neurosci. 13, 362–365. 10.1016/0166-2236(90)90017-5 1699323

[B17] Ferreira T. A. Blackman A. V. Oyrer J. Jayabal S. Chung A. J. Watt A. J.. (2014). Neuronal morphometry directly from bitmap images. Nat. Methods 11, 982–984. 10.1038/nmeth.3125 25264773 PMC5271921

[B18] Ferretti P. (2011). Is there a relationship between adult neurogenesis and neuron generation following injury across evolution? Eur. J. Neurosci. 34, 951–962. 10.1111/j.1460-9568.2011.07833.x 21929627

[B19] Frontera J. L. Cervino A. S. Jungblut L. D. Paz D. A. (2015). Brain-derived neurotrophic factor (BDNF) expression in normal and regenerating olfactory epithelium of *Xenopus laevis*. Ann. Anat. 198, 41–48. 10.1016/j.aanat.2014.10.010 25488259

[B20] Frontera J. L. Raices M. Cervino A. S. Pozzi A. G. Paz D. A. (2016). Neural regeneration dynamics of *Xenopus laevis* olfactory epithelium after zinc sulfate-induced damage. J. Chem. Neuroanat. 77, 1–9. 10.1016/j.jchemneu.2016.02.003 27012180

[B21] Gliem S. Syed A. S. Sansone A. Kludt E. Tantalaki E. Hassenklöver T.. (2013). Bimodal processing of olfactory information in an amphibian nose: odor responses segregate into a medial and a lateral stream. Cell. Mol. Life Sci. 70, 1965–1984. 10.1007/s00018-012-1226-8 23269434 PMC3656224

[B22] Graziadei P. P. (1973). Cell dynamics in the olfactory mucosa. Tissue Cell 5, 113–131. 10.1016/s0040-8166(73)80010-2 4540384

[B23] Graziadei P. P. C. DeHan R. S. (1973). Neuronal regeneration in frog olfactory system. J. Cell Biol. 59, 525–530. 10.1083/jcb.59.2.525 4548906 PMC2109092

[B24] Graziadei P. P. Graziadei G. (1979). Neurogenesis and neuron regeneration in the olfactory system of mammals. I. Morphological aspects of differentiation and structural organization of the olfactory sensory neurons. J. Neurocytol. 8, 1–18. 10.1007/bf01206454 438867

[B25] Graziadei P. P. Metcalf J. (1971). Autoradiographic and ultrastructural observations on the frog’s olfactory mucosa. Z. Zellforsch. Mikrosk. Anat. 116, 305–318. 10.1007/bf00330630 4931733

[B26] Gudziol V. Hoenck I. Landis B. Podlesek D. Bayn M. Hummel T. (2014). The impact and prospect of traumatic brain injury on olfactory function: a cross-sectional and prospective study. Eur. Arch. Otorhinolaryngol. 271, 1533–1540. 10.1007/s00405-013-2687-6 24071856

[B27] Haas K. Jensen K. Sin W. C. Foa L. Cline H. T. (2002). Targeted electroporation in *Xenopus* tadpoles *in vivo*—from single cells to the entire brain. Differentiation 70, 148–154. 10.1046/j.1432-0436.2002.700404.x 12147134

[B29] Hassenklöver T. Kurtanska S. Bartoszek I. Junek S. Schild D. Manzini I. (2008). Nucleotide-induced Ca^2+^ signaling in sustentacular supporting cells of the olfactory epithelium. Glia 56, 1614–1624. 10.1002/glia.20714 18551628

[B28] Hassenklöver T. Manzini I. (2014). The olfactory system as a model to study axonal growth patterns and morphology *in vivo*. J. Vis. Exp. 92:e52143. 10.3791/52143 25406975 PMC4353389

[B30] Hassenklöver T. Schwartz P. Schild D. Manzini I. (2009). Purinergic signaling regulates cell proliferation of olfactory epithelium progenitors. Stem Cells 27, 2022–2031. 10.1002/stem.126 19544419

[B31] Huard J. M. Youngentob S. L. Goldstein B. J. Luskin M. B. Schwob J. E. (1998). Adult olfactory epithelium contains multipotent progenitors that give rise to neurons and non-neural cells. J. Comp. Neurol. 400, 469–486. 10.1002/(sici)1096-9861(19981102)400:4<469::aid-cne3>3.0.co;2-8 9786409

[B32] Imai T. Sakano H. (2011). Axon-axon interactions in neuronal circuit assembly: lessons from olfactory map formation. Eur. J. Neurosci. 34, 1647–1654. 10.1111/j.1460-9568.2011.07817.x 22103421

[B33] Imamura F. Greer C. A. (2009). Dendritic branching of olfactory bulb mitral and tufted cells: regulation by TrkB. PLoS One 4:e6729. 10.1371/journal.pone.0006729 19707543 PMC2727791

[B35] Jia C. Doherty J. P. Crudgington S. Hegg C. C. (2009). Activation of purinergic receptors induces proliferation and neuronal differentiation in Swiss Webster mouse olfactory epithelium. Neuroscience 163, 120–128. 10.1016/j.neuroscience.2009.06.040 19555741 PMC2728178

[B36] Jia C. Hayoz S. Hutch C. R. Iqbal T. R. Pooley A. E. Hegg C. C. (2013). An IP3R3- and NPY-expressing microvillous cell mediates tissue homeostasis and regeneration in the mouse olfactory epithelium. PLoS One 8:e58668. 10.1371/journal.pone.0058668 23516531 PMC3596314

[B34] Jia C. Hegg C. C. (2010). NPY mediates ATP-induced neuroproliferation in adult mouse olfactory epithelium. Neurobiol. Dis. 38, 405–413. 10.1016/j.nbd.2010.02.013 20211262 PMC2862820

[B37] Junek S. Chen T.-W. Alevra M. Schild D. (2009). Activity correlation imaging: visualizing function and structure of neuronal populations. Biophys. J. 96, 3801–3809. 10.1016/j.bpj.2008.12.3962 19413986 PMC2711456

[B38] Kaplan M. Hinds J. (1977). Neurogenesis in the adult rat: electron microscopic analysis of light radioautographs. Science 197, 1092–1094. 10.1126/science.887941 887941

[B39] Kauffman S. (1968). Lengthening of the generation cycle during embryonic differentiation of the mouse neural tube. Exp. Cell Res. 49, 420–424. 10.1016/0014-4827(68)90191-2 5760443

[B40] Kobayashi M. Costanzo R. M. (2009). Olfactory nerve recovery following mild and severe injury and the efficacy of dexamethasone treatment. Chem. Senses 34, 573–580. 10.1093/chemse/bjp038 19578153 PMC2728832

[B41] Kosaka T. Kosaka K. (2016). Neuronal organization of the main olfactory bulb revisited. Anat. Sci. Int. 91, 115–127. 10.1007/s12565-015-0309-7 26514846

[B42] Leung C. T. Coulombe P. A. Reed R. R. (2007). Contribution of olfactory neural stem cells to tissue maintenance and regeneration. Nat. Neurosci. 10, 720–726. 10.1038/nn1882 17468753

[B43] Lim D. A. Alvarez-Buylla A. (2016). The adult ventricular-subventricular zone (V-SVZ) and olfactory bulb (OB) neurogenesis. Cold Spring Harb. Perspect. Biol. 8:a018820. 10.1101/cshperspect.a018820 27048191 PMC4852803

[B44] Malun D. Brunjes P. C. (1996). Development of olfactory glomeruli: temporal and spatial interactions between olfactory receptor axons and mitral cells in opossums and rats. J. Comp. Neurol. 368, 1–16. 10.1002/(sici)1096-9861(19960422)368:1<1::aid-cne1>3.0.co;2-7 8725290

[B45] Manzini I. (2015). From neurogenesis to neuronal regeneration: the amphibian olfactory system as a model to visualize neuronal development *in vivo*. Neural Regen. Res. 10, 872–874. 10.4103/1673-5374.158334 26199593 PMC4498338

[B46] Matthews M. Powell T. (1962). Some observations on transneuronal cell degeneration in the olfactory bulb of the rabbit. J. Anat. 96, 89–102. 14471407 PMC1244175

[B47] Meisami E. Safari L. (1981). A quantitative study of the effects of early unilateral olfactory deprivation on the number and distribution of mitral and tufted cells and of glomeruli in the rat olfactory bulb. Brain Res. 221, 81–107. 10.1016/0006-8993(81)91065-9 7272762

[B48] Miragall F. Graziadei G. A. M. (1982). Experimental studies on the olfactory marker protein. II. Appearance of the olfactory marker protein during differentiation of the olfactory sensory neurons of mouse: an immunohistochemical and autoradiographic study. Brain Res. 239, 245–250. 10.1016/0006-8993(82)90846-0 7046875

[B49] Mizrahi A. Katz L. C. (2003). Dendritic stability in the adult olfactory bulb. Nat. Neurosci. 6, 1201–1207. 10.1038/nn1133 14528309

[B50] Mouret A. Lepousez G. Gras J. Gabellec M.-M. Lledo P.-M. (2009). Turnover of newborn olfactory bulb neurons optimizes olfaction. J. Neurosci. 29, 12302–12314. 10.1523/jneurosci.3383-09.2009 19793989 PMC6666148

[B51] Murai A. Iwata R. Fujimoto S. Aihara S. Tsuboi A. Muroyama Y.. (2016). Distorted coarse axon targeting and reduced dendrite connectivity underlie dysosmia after olfactory axon injury. eNeuro 3:ENEURO.0242-16.2016. 10.1523/eneuro.0242-16.2016 27785463 PMC5066264

[B52] Nagayama S. Homma R. Imamura F. (2014). Neuronal organization of olfactory bulb circuits. Front. Neural Circuits 8:98. 10.3389/fncir.2014.00098 25232305 PMC4153298

[B54] Nezlin L. P. Heermann S. Schild D. Rössler W. (2003). Organization of glomeruli in the main olfactory bulb of *Xenopus laevis* tadpoles. J. Comp. Neurol. 464, 257–268. 10.1002/cne.10709 12900923

[B53] Nezlin L. P. Schild D. (2000). Structure of the olfactory bulb in tadpoles of *Xenopus laevis*. Cell Tissue Res. 302, 21–29. 10.1007/s004410000208 11079712

[B55] Nieuwkoop P. D. Faber J. (1994). Normal Table of Xenopus laevis (Daudin). New York, NY: Garland Publishing Inc.

[B56] Nishizumi H. Sakano H. (2015). Developmental regulation of neural map formation in the mouse olfactory system. Dev. Neurobiol. 75, 594–607. 10.1002/dneu.22268 25649346

[B57] Otsu N. (1979). A threshold selection method from gray-level histograms. IEEE Trans. Syst. Man Cybern. 9, 62–66. 10.1109/tsmc.1979.4310076

[B58] Preibisch S. Saalfeld S. Tomancak P. (2009). Globally optimal stitching of tiled 3D microscopic image acquisitions. Bioinformatics 25, 1463–1465. 10.1093/bioinformatics/btp184 19346324 PMC2682522

[B59] Raices M. Jungblut L. D. Paz D. A. Pozzi A. G. (2015). Cytotoxic effect of 5-bromo-2-deoxyuridine on olfactory epithelium. MOJ Anat. Physiol. 1:00023 10.15406/mojap.2015.01.00023

[B60] Roskams A. J. Bethel M. Hurt K. Ronnett G. V. (1996). Sequential expression of Trks A, B, and C in the regenerating olfactory neuroepithelium. J. Neurosci. 16, 1294–1307. 8778281 10.1523/JNEUROSCI.16-04-01294.1996 PMC6578567

[B61] Schindelin J. Arganda-Carreras I. Frise E. Kaynig V. Longair M. Pietzsch T.. (2012). Fiji: an open-source platform for biological-image analysis. Nat. Methods 9, 676–682. 10.1038/nmeth.2019 22743772 PMC3855844

[B62] Schwob J. E. (2002). Neural regeneration and the peripheral olfactory system. Anat. Rec. 269, 33–49. 10.1002/ar.10047 11891623

[B63] Sholl D. (1953). Dendritic organization in the neurons of the visual and motor cortices of the cat. J. Anat. 87, 387–406. 13117757 PMC1244622

[B64] Syed A. S. Sansone A. Hassenklöver T. Manzini I. Korsching S. I. (2016). Coordinated shift of olfactory amino acid responses and V2R expression to an amphibian water nose during metamorphosis. Cell. Mol. Life Sci. 74, 1711–1719. 10.1007/s00018-016-2437-1 27990576 PMC11107701

[B65] Tavosanis G. (2012). Dendritic structural plasticity. Dev. Neurobiol. 72, 73–86. 10.1002/dneu.20951 21761575

[B66] Terni B. Pacciolla P. Masanas H. Gorostiza P. Llobet A. (2017). Tight temporal coupling between synaptic rewiring of olfactory glomeruli and the emergence of odor-guided behavior in *Xenopus* tadpoles. J. Comp. Neurol. 525, 3769–3783. 10.1002/cne.24303 28815589

[B67] Thompson C. K. Brenowitz E. A. (2010). Neuroprotective effects of testosterone in a naturally occurring model of neurodegeneration in the adult avian song control system. J. Comp. Neurol. 518, 4760–4770. 10.1002/cne.22486 20963827 PMC2963470

[B68] Yoshino J. Tochinai S. (2006). Functional regeneration of the olfactory bulb requires reconnection to the olfactory nerve in *Xenopus larvae*. Dev. Growth Differ. 48, 15–24. 10.1111/j.1440-169x.2006.00840.x 16466389

[B69] Yu C. R. Wu Y. (2017). Regeneration and rewiring of rodent olfactory sensory neurons. Exp. Neurol. 287, 395–408. 10.1016/j.expneurol.2016.06.001 27264358

